# The Clinical Significance of Cancer-Associated Fibroblasts Classification in Non-Small Cell Lung Cancer

**DOI:** 10.3390/cells13221909

**Published:** 2024-11-19

**Authors:** Kostas A. Papavassiliou, Amalia A. Sofianidi, Vassiliki A. Gogou, Athanasios G. Papavassiliou

**Affiliations:** 1 First University Department of Respiratory Medicine, ‘Sotiria’ Chest Hospital, Medical School, National and Kapodistrian University of Athens, 11527 Athens, Greece; konpapav@med.uoa.gr (K.A.P.); gogvanessa@gmail.com (V.A.G.); 2 Department of Biological Chemistry, Medical School, National and Kapodistrian University of Athens, 11527 Athens, Greece; amsof.00@gmail.com

Malignant cells flourish within a specialized environment known as the tumor microenvironment (TME). This dynamic ecosystem is rich in various components, including immune cells, stromal cells, blood vessels, and the extracellular matrix (ECM) [1]. Lately, cancer investigators have drawn closer attention to a particular resident of the TME, namely fibroblasts [2,3]. The role of fibroblasts is not a new discovery; Harold Dvorak famously described tumors as “wounds that do not heal” as early as 1986, accentuating the relentless and irreversible activation of these cells within the TME [4]. These cells, now known as cancer-associated fibroblasts (CAFs), play a crucial role in shaping the TME and supporting cancer growth, making them a key focus of current cancer research [3]. In non-small cell lung cancer (NSCLC) specifically, CAFs are increasingly recognized as pivotal drivers of tumor initiation, progression [5], and immune suppression [6]. This emerging understanding highlights the importance of molecularly classifying CAF subtypes, as these distinctions could play a decisive role in predicting prognosis, optimizing therapeutic responses, and improving clinical outcomes for NSCLC patients.

To molecularly classify CAFs, we must first clarify their precise cellular origins. CAFs can arise from quiescent fibroblasts residing in the stroma, which, under specific tumorigenic conditions, transform into activated CAFs, adopting a myofibroblast-like phenotype and expressing alpha-smooth muscle actin (a-SMA) [7]. Other cell types have also been identified as potential sources of CAFs, including mesenchymal stem cells, stellate cells, epithelial cells, pericytes, and even adipocytes [7]. Notably, one of the most striking features of CAFs is their dual role within the TME; depending on the conditions, they can either promote or inhibit tumor growth, acting as a double-edged sword in NSCLC progression [8]. The functional diversity of CAFs has led researchers to propose that it is not merely the tumor environment but rather the inherent molecular heterogeneity of CAFs that determines their role in fostering or hampering tumorigenesis. Preclinical studies in gastrointestinal cancer models have shown that certain CAF subpopulations can even exhibit tumor-suppressive effects to a degree [9,10]. Whether similar subpopulations with tumor-suppressing capabilities exist in lung cancer models remains an area of active investigation. The sole reference to a tumor-suppressing role of CAFs in NSCLC comes from the study of Cords et al., who identified a specific CAF subtype that mediates its antitumor effects via interferon signaling [11].

The most significant contribution of CAF subtyping to NSCLC lies in its ability to predict patient prognosis. In a comprehensive study involving over 1000 NSCLC patients, Cords et al. revealed that different CAF phenotypes not only occupy distinct spatial regions within the TME but also serve as independent prognostic factors for patient outcomes [11]. Patient groups with favorable prognoses showed enrichment in fibroblast markers like α-SMA and cluster of differentiation 34 (CD34: a transmembrane, cell surface phosphoglycoprotein functioning as a cell–cell adhesion factor) or displayed activity through interferon signaling. These particular CAF subtypes were often located in the vicinity of blood vessels, suggesting a close association with immune cell infiltration. In contrast, CAFs linked to poorer prognoses were frequently found within hypoxic TMEs or exhibited markers such as transforming growth factor beta (TGF-β); CD10 (a type II transmembrane, cell surface glycoprotein belonging to peptidase M13 family, necessary for angiogenesis and so forth), and CD73 (known as ecto-5′-nucleotidase, one of the key enzymes in the purinergic signaling pathway responsible for the generation of immune suppressive adenosine) [11]. Conflicting with that, findings in small cell lung cancer (SCLC) cell lines revealed that elevated levels of secreted TGF-β1 correlate with longer overall survival and improved prognosis [12]. This disparity underscores the significant heterogeneity among lung cancer subtypes: the same CAF phenotypes can lead to markedly different survival outcomes. Such findings also emphasize the importance of personalized research approaches in the TME of lung cancer patients. Notably, recent research identified a novel CAF subtype, termed CAF-S5, which expresses fibroblast activation protein (FAP: a type II transmembrane serine protease best known for its heightened expression in tumor stroma) and podoplanin (PDPN: a small, mucin-like transmembrane glycoprotein with a broad spectrum of functions including tumorigenesis and metastasis) but lacks α-SMA. This subtype has been linked to worse clinical outcomes in NSCLC patients, providing new insights into CAF-related prognostic markers [13].

Molecular classification of CAFs in NSCLC tissue could also enhance the prediction of patient responses to various treatment options. Interestingly, high CD10 expression in NSCLC CAFs has been linked to resistance to chemotherapy [11,14], underlining the potential of CAF profiling in guiding therapeutic strategies. Feng et al. identified a CAF subtype that produces the tryptophan metabolite kynurenine, which has been associated with resistance to epidermal growth factor receptor (EGFR) tyrosine kinase inhibitor (TKI) treatments in NSCLC cell lines [15]. Further supporting this observation, CD200 (a membrane glycoprotein that mediates suppression of T-cell-mediated immune responses) positive CAFs were found to increase cancer cell sensitivity to the EGFR TKI gefitinib in lung adenocarcinoma preclinical models [16]. Moreover, CAFs in NSCLC have been demonstrated to augment the sensitivity of malignant cells to EGFR TKIs, through the secretion of insulin-like growth factor (IGF) and IGF-binding proteins (IGFBPs) [17]. Regarding immunotherapy options, CAF subtypes associated with immune enrichment in NSCLC patients—such as the interferon-producing subtype identified by Cords et al. [11]—may lead to improved response rates to immunotherapy. Although it involves a different lung cancer subtype, CAFs secreting TGF-β have been found to enhance radiosensitivity in SCLC cell lines [12]. This observation suggests that phenotypic classification of CAFs in NSCLC could also serve as a predictor of response to radiation therapy.

To date, CAF-based therapeutic strategies are under development in breast, pancreatic, lung, colorectal and prostate cancer and melanoma [8]. The functional heterogeneity of CAFs [18] complicates research towards effective targeting of this central component of the TME. Delving deeper into the molecular characteristics of CAFs and being able to efficiently subtype different CAF phenotypes could pave the way for more effectual and personalized cancer treatment options, not only in NSCLC but in many other types of malignancy. Phenotypic plasticity, as described by Hanahan et al. in 2022 [19], is also a hallmark of CAFs, and it was recently confirmed in pancreatic cancer [20]. Further studies could also verify this hallmark in NSCLC—possibly driven by the expression of specific biomarkers—making the induction of CAF conversion a beneficial treatment modality alongside directly targeting CAFs. By targeting CAFs with known roles in promoting tumor growth, metastasis, or immune evasion, therapies may be developed that hinder these processes, potentially ameliorating NSCLC treatment outcomes and patients’ overall prognosis.
